# CRISPR-Cpf1-Mediated Gene-Editing System Based on a Single Bidirectional Promoter

**DOI:** 10.3390/ijms27104162

**Published:** 2026-05-07

**Authors:** Soomin Kim, Gyeong-Nam Kim, Yeon-Ju Jeong, Jeongin Cho, Mingyo Jang, Jinpyo Hong, Young Hoon Sung

**Affiliations:** 1 Department of Medical Science, Asan Medical Institute of Convergence Science and Technology, University of Ulsan College of Medicine, Seoul 05505, Republic of Korea; lucykim0214@naver.com (S.K.);; 2 Department of Convergence Medicine, Convergence Medicine Research Center, Biomedical Research Center, Asan Institute for Life Sciences, Asan Medical Center, Seoul 05505, Republic of Korea; 3 Department of Cell and Genetic Engineering, University of Ulsan College of Medicine, Seoul 05505, Republic of Korea

**Keywords:** CRISPR–AsCpf1, genome editing, bidirectional H1 promoter, AAV delivery

## Abstract

Recent advances in gene therapy have highlighted the potential of CRISPR-based gene-editing systems combined with adeno-associated virus (AAV) vectors. However, the limited packaging capacity of AAV remains a significant challenge for the simultaneous expression of Cas effector proteins and guide RNAs within a single vector. To address this limitation, we developed a compact AAV vector that enables the co-expression of *Acidaminococcus* sp. Cas12a (AsCpf1) and CRISPR RNAs (crRNAs) using a single bidirectional promoter derived from the mouse H1 promoter. Our single bidirectional H1 promoter supported indel formation comparable to that achieved by dual-promoter systems and facilitated scalable genome editing with single-, dual-, and triple-target configurations. Genome editing was successfully accomplished both in vitro and in vivo following AAV delivery. This study shows that our engineered compact AAV vector platform is capable of simultaneously delivering AsCpf1 and multiplexed crRNAs.

## 1. Introduction

The CRISPR–Cas system is a widely used tool for genome engineering; however, its clinical translation remains constrained by the large size of Cas effector proteins. These constraints are particularly restrictive for adeno-associated virus (AAV) delivery, as the ~4.7 kb packaging capacity severely limits therapeutic vector design. Consequently, these limitations have driven the development of compact CRISPR–Cas systems [1,2]. While these systems offer reduced vector size, they may exhibit limitations in editing efficiency or target specificity, requiring alternative strategies that balance compactness and functional robustness.

Cas12a (Cpf1), a Class 2, Type V CRISPR nuclease, offers several advantages over Cas9, including its requirement for only a single crRNA, intrinsic RNase activity that enables autonomous crRNA processing, and recognition of T-rich PAM sequences, which expand the range of targetable genomic loci [3]. Cpf1 orthologues also support high-fidelity multiplex genome editing, making them attractive candidates for therapeutic applications [4]. Despite these advantages, single-vector AAV delivery of Cpf1 remains challenging because conventional dual-promoter architectures for nuclease and crRNA expression often exceed the AAV packaging limit. Therefore, a compact promoter architecture capable of driving both nuclease and crRNA expression is critical for efficient single-vector AAV delivery. In AAV-based CRISPR systems, promoter selection plays a critical role in balancing between the expression level and vector size. RNA polymerase III (Pol III) promoters such as U6 and H1 are commonly used for guide RNA expression, whereas RNA polymerase II (Pol II) promoters such as CMV or EFS are typically used to drive the expression of CRISPR nucleases [5,6]. However, dual-promoter architectures increase vector size and thus limit their compatibility with AAV delivery. Therefore, bidirectional promoters provide a compact alternative by enabling coordinated expression of coding and non-coding transcripts from a single regulatory element [7,8].

The human H1 promoter is well characterized for precise RNA transcription by RNA polymerase III and has been widely used to express guide RNAs for Cas9-mediated genome editing [5]. Notably, the H1 promoter can also support RNA polymerase II–dependent transcription [6]. Exploiting this property, Gao et al. positioned guide RNA and Cas9 expression cassettes in proximity and observed robust indel formation in cultured cells in vitro [9]. Moreover, the endogenous H1 promoter bidirectionally drives transcription of H1 RNA and the *Parp2* gene in opposite orientations [7] and has previously been used to co-express shRNAs and protein-coding sequences [8].

In this study, we established a compact AAV platform utilizing the bidirectional activity of the H1 promoter, enabling the simultaneous expression of Cpf1 and its crRNAs from a single promoter. This design provides a more compact configuration and may allow more consistent guide RNA expression compared to conventional Pol II-driven crRNA array systems, suitable for AAV applications. We demonstrate that this system supports efficient genome editing both in vitro and in vivo.

## 2. Results

### 2.1. Optimization of the Bidirectional mH1 Promoter for Co-Expression of AsCpf1 and crRNA

**Figure 1:**
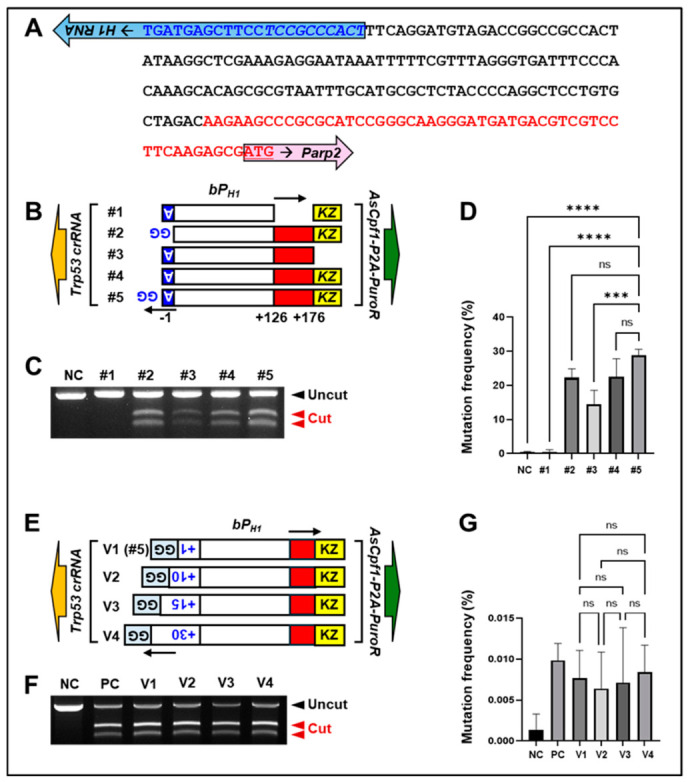
Optimization of bidirectional H1 promoter engineering for crRNA and AsCpf1 expression and indel generation. (**A**) Sequence of the mouse H1 promoter driving the expression of *Parp2*. Black, core promoter sequence of the bidirectional H1 promoter; blue, transcription initiation site of H1 RNA; red, 5′ UTR of *Parp2* mRNA. (**B**) Five variant lentiviral constructs of the bidirectional H1 promoters designed to enhance *Trp53* crRNA and AsCpf1 expression. Blue, transcription initiation site (±A ± GG motif) of *Trp53* crRNA; red box: 5′ UTR of *Parp2* mRNA; yellow box: Kozak sequence (KZ) of AsCpf1-P2A-PuroR. (**C**) T7E1 assay of genomic DNA from puromycin-selected NIH3T3 cells. NC, non-infected cells as a negative control. Black arrowhead, uncut DNA fragments by T7E1; red arrowhead, cut DNA fragments by T7E1. (**D**) Statistical analysis of Figure 1C. Mutation frequency (%) was calculated from the fraction of cleaved DNA using Image J software version 1.54f. Statistical analysis was performed using one-way ANOVA followed by Tukey’s multiple comparisons test. Data are presented as mean ± SD from three independent experiments (*n* = 3). *** *p* < 0.001, **** *p* < 0.0001; ns, not significant. (**E**) Structure of a series of modified bidirectional H1 promoters. Numbers in blue indicate number of nucleotide sequences added. The newly added sequences to each construct are underlined as follows: V1 (5′-AGG-3′), V2 (5′-AGT GGG CGG AGG-3′), V3 (5′-AGT GGG CGG AGG AAG GG-3′), V4 (5′-AGT GGG CGG AGG AAG CTC ATC AGC GGG GCC GG-3′). Red box, 5′ UTR of *Parp2* mRNA; yellow box, Kozak sequence (KZ). (**F**) T7E1 assays using genomic DNA samples from NIH3T3 cells transduced with lentiviruses harboring H1 promoter variants. NC, non-infected cells as a negative control; PC, gene editing driven by lentivirus with two promoters (EFS/U6) as a positive control. Black arrowhead, uncut DNA fragments; red arrowhead, DNA fragments cut by T7E1. Indel frequency was estimated by densitometric analysis of T7E1 assay results. A representative result of three independent experiments. (**G**) Statistical analysis of Figure 1E. Mutation frequency (%) was calculated from the fraction of cleaved DNA using Image J software version 1.54f. Statistical analysis was performed using one-way ANOVA followed by Tukey’s multiple comparisons test. Data are presented as mean ± SD from three independent experiments (*n* = 3). *** *p* < 0.001, **** *p* < 0.0001; ns, not significant.

To develop an AAV vector capable of simultaneously expressing both AsCpf1 (*Acidaminococcus* sp. *Cas12a*) and its crRNAs, we utilized the mouse H1 promoter, which is significantly shorter than the human H1 promoter [10,11]. Based on the mouse H1 promoter sequence (Figure 1A), we designed and tested five variants to identify an optimized promoter configuration. These variants differed in the 5′ GG motif at the H1 RNA transcription start site, the *Parp2* 5′ UTR, and the Kozak sequence upstream of AsCpf1 (Figure 1B and Figure S1). The promoters were subcloned into a lentiviral vector containing the AsCpf1-P2A-PuroR cassette and a *Trp53* crRNA sequence.

To assess indel activity, lentiviruses were produced in HEK293T cells and used to infect NIH3T3 cells. Following lentiviral infection, cells transduced with promoter #1, which lacks the *Parp2* 5′ UTR, exhibited extensive cell death even after puromycin selection, whereas cells expressing the other promoter variants survived puromycin treatment (Figure S2). This suggests that inclusion of the *Parp2* 5′ UTR is critical for maintaining appropriate AsCpf1 expression and cellular viability. After puromycin selection, genomic DNA was extracted and analyzed for indels using a T7E1 assay (Figure 1C). Among the variants, promoter #5 exhibited the highest indel activities over the other constructs (Figure 1D).

To further investigate the impact of 5′ UTR length, we constructed three additional variant vectors based on promoter #5 (Figure 1E). After lentiviral transduction of NIH3T3 cells and puromycin selection, genomic DNA was extracted, and indel frequencies were assessed using T7E1 assays (Figure 1F). No apparent differences in genome-editing efficiency were observed among these variants (Figure 1G); we selected the version #2 vector for subsequent experiments. These results indicate that a 5′ di-guanosine (GG) sequence at the crRNA transcription initiation site, the 5′ UTR of *Parp2*, and the Kozak sequence of the AsCpf1-P2A-Puromycin resistance gene expression cassette are critical for the enhanced genome-editing efficiency of the H1 promoter-mediated CRISPR-Cpf1 expression vector.

### 2.2. Confirmation of Bidirectional H1 Promoter Systems for CRISPR-Cpf1 Expression

**Figure 2:**
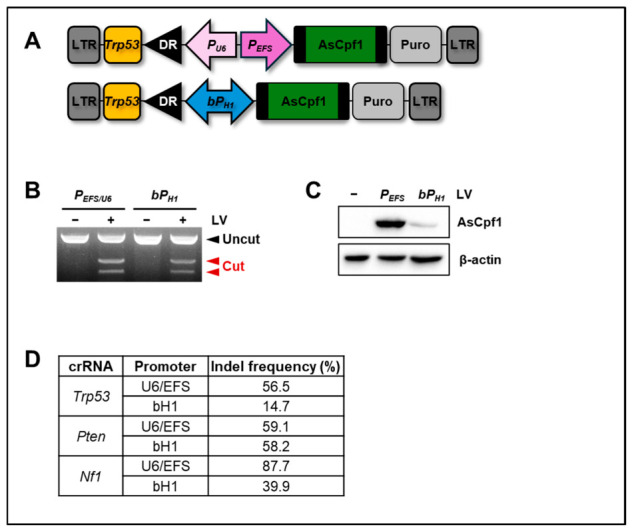
Confirmation of bidirectional H1 promoter systems for CRISPR-AsCpf1 expression. (**A**) Schematic diagrams of two CRISPR-AsCpf1 constructs. Top panel: AsCpf1-P2A-PuroR driven by the EFS promoter and *Trp53* crRNA driven by the U6 promoter. Bottom panel: Bidirectional promoter driving both AsCpf1-P2A-PuroR and *Trp53* crRNA. DR, direct repeat. (**B**) T7E1 assay for indel frequency analysis. NIH3T3 cells were infected with lentiviruses carrying the two constructs. After puromycin selection, genomic DNA was extracted and subjected to the T7E1 assay. Black arrowhead, uncut DNA fragments; red arrowhead, DNA fragments cleaved by T7E1. (**C**) Western blot analysis of AsCpf1 expression. Cell lysates from puromycin-selected cells were analyzed by Western blotting. β-actin was used as a loading control. (**D**) Deep sequencing analysis of indel frequencies. Indel frequencies at the *Trp53*, *Pten*, and *Nf1* loci were compared between the dual U6/EFS promoter system and the bidirectional promoter system using deep sequencing. A representative result of two independent experiments.

To confirm the performance of the single-promoter H1 system (177 bp), we targeted the *Trp53* gene in NIH3T3 cells with the 501 bp conventional dual-promoter U6/EFS system (Figure 2A). The H1 promoter system efficiently induced indel formation at the *Trp53* locus, producing indel frequencies that were slightly lower but comparable to those achieved by the dual-promoter U6/EFS system (Figure 2B). To determine whether differences in indel efficiency correlated with AsCpf1 expression levels, we analyzed AsCpf1 protein abundance by Western blotting. As expected, the EFS promoter drove higher AsCpf1 expression than the H1 promoter system (Figure 2C). We next assessed the versatility of the H1 promoter system by measuring genome-editing activity at multiple loci, including *Trp53*, *Pten*, and *Nf1* (Figure 2D). Indel frequencies at the *Pten* locus were comparable between the single-promoter and control systems. Although indel frequencies at the *Trp53* and *Nf1* loci were modestly reduced under the single-promoter condition, genome editing was consistently observed across all target sites. These results support that the H1 promoter can drive the simultaneous expression of both AsCpf1 and crRNAs at levels sufficient to induce indel mutations at multiple genomic loci.

### 2.3. Functionality of the Single-Vector AAV–Mediated Genome-Editing System In Vitro and In Vivo

**Figure 3:**
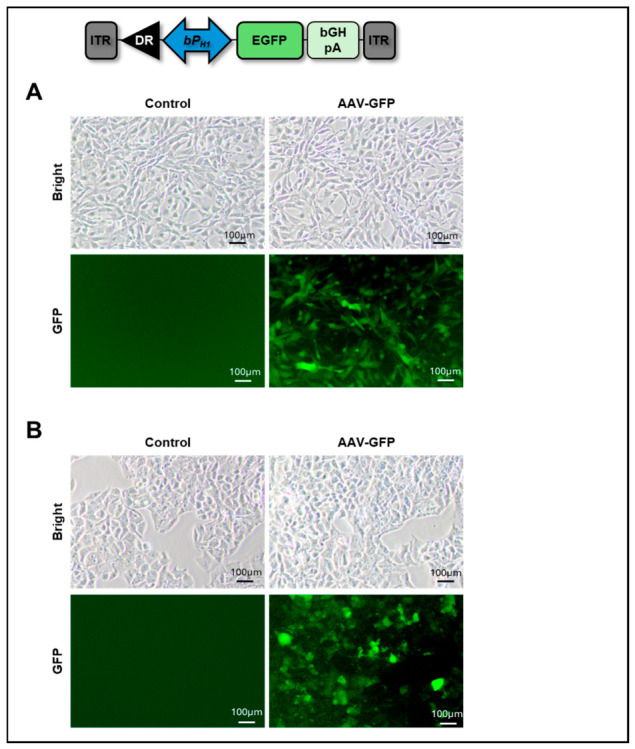
Mouse H1 promoter-driven EGFP expression in mouse and human cells. An AAV-DJ vector encoding EGFP under the control of a bidirectional mouse H1 promoter was used. EGFP expression was examined 3 days post-infection using a fluorescence microscope. (**A**) EGFP expression in mouse NIH3T3 cells. (**B**) EGFP expression in human HepG2 cells. Representative results of three independent experiments.

To evaluate the functionality of the H1 promoter within the AAV system, we first constructed an AAV-DJ vector expressing EGFP under the control of this promoter before integrating the CRISPR-AsCpf1 AAV system. NIH3T3 and HepG2 cells were transduced at an MOI of approximately 1 × 10^4^ genome copies per cell. Two days after AAV transduction, EGFP expression was readily detected in both NIH3T3 and HepG2 cells (Figure 3). EGFP expression was stably detectable at 3 days post-infection (Figure 3). While transduction efficiency was not quantitatively assessed, the robust EGFP signals suggest effective viral transduction under these conditions.

**Figure 4:**
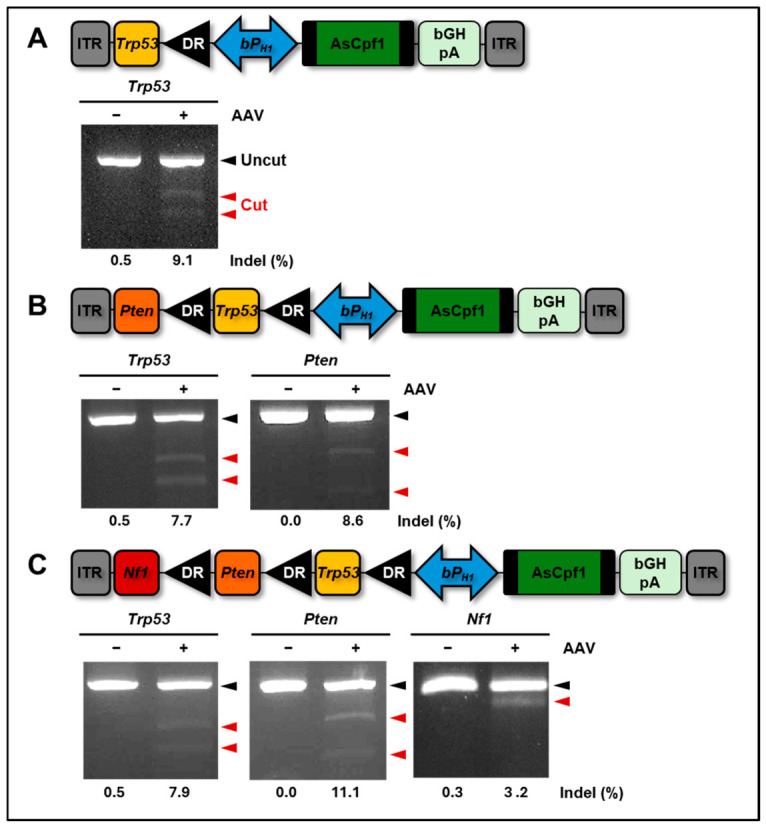
Multiplex genome editing using the CRISPR-AsCpf1 AAV system with a bidirectional H1 promoter. DR, direct repeat. AAV vectors targeting the *Trp53* locus (**A**), *Trp53* and *Pten* loci (**B**), and *Trp53*, *Pten*, and *Nf1* loci (**C**) were infected into primary MEF cells. After 3 days, indel mutants were also assayed by T7E1 assays, and then their frequencies (%) were determined by deep sequencing analyses using genomic DNA extracted from AAV-infected cells. Black arrowheads, uncut DNA fragments; red arrowheads, DNA fragments cleaved by T7E1; indel (%), the results of deep sequencing analyses. A representative result of three independent experiments.

Subsequently, we constructed a CRISPR–AsCpf1 AAV system driven by the H1 promoter and evaluated its genome-editing activity in primary wild-type MEFs. The *Trp53*-specific AAV induced detectable indel mutations (Figure 4A). Furthermore, application of AAV particles expressing multiplex crRNAs simultaneously targeting both the *Trp53* and *Pten* loci resulted in comparable indel frequencies (Figure 4B).

Finally, we tested whether this AAV vector could target three genes simultaneously and observed the highest editing efficiency at the *Pten* locus, with relatively low indel frequencies at the *Trp53* and *Nf1* loci (Figure 4C). We compared the SV40 and bovine growth hormone (bGH) polyadenylation signals to determine whether poly(A) signal affects indel frequency. No apparent differences in indel frequencies were observed, suggesting that the shorter SV40 polyadenylation signal can be used to accommodate additional genetic elements without compromising editing efficiency (Figure S3).

**Figure 5:**
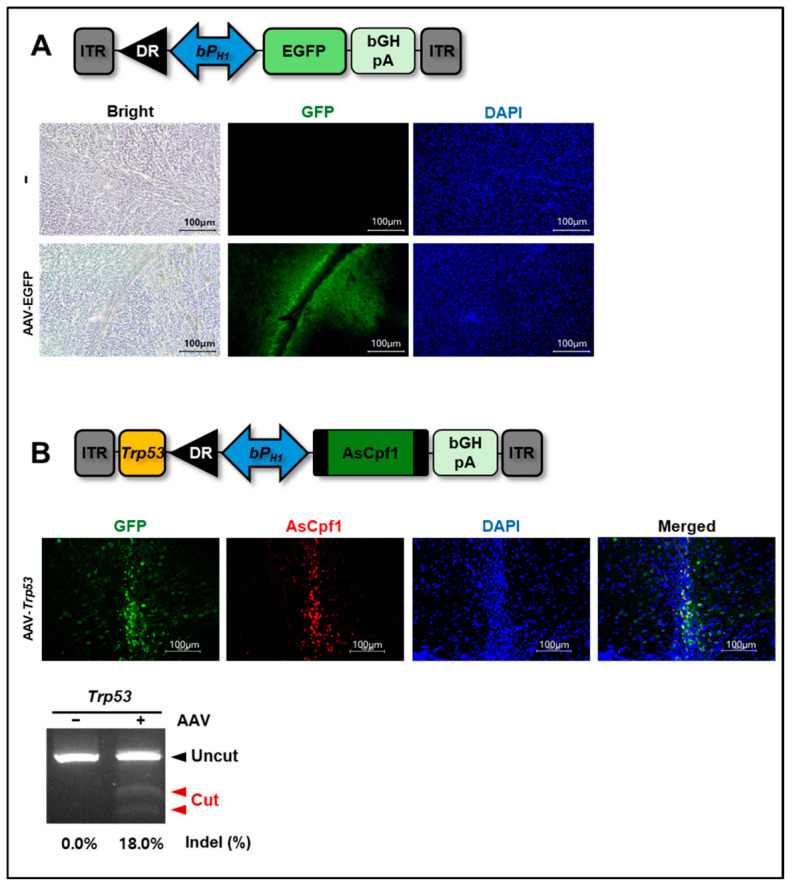
In vivo genome editing using the CRISPR-AsCpf1 AAV system driven by a bidirectional H1 promoter. DR, direct repeat. (**A**) EGFP expression driven by the bidirectional H1 promoter in the striatum. An AAV vector encoding EGFP under this promoter was delivered to the right striatum via stereotaxic surgery (coordinates: AP +0.2, ML +2.0, DV −3.5). EGFP signals in the striatum were examined by fluorescence microscopy 21 days post-injection. (**B**) Genome editing in the striatum by CRISPR-AsCpf1 AAV targeting the *Trp53* locus. A representative result of two independent experiments. AsCpf1 expression driven by the bidirectional H1 promoter was confirmed by immunohistochemistry using anti-AsCpf1 antibodies in an EGFP-positive slide. Indel mutants were also assayed by T7E1 assays, and then their frequencies (%) were determined by deep sequencing analyses using genomic DNA extracted from AAV-infected, EGFP-positive tissues. Black arrowheads, uncut DNA fragments; red arrowheads, DNA fragments cleaved by T7E1, indicating indel formation; Indel (%), the results of deep sequencing analyses.

These findings show that the H1 promoter-driven CRISPR-AsCpf1 AAV system is effective for genome editing. To evaluate the in vivo efficacy of our system, we simultaneously injected an AAV encoding EGFP (3992 bp) and an AAV targeting the *Trp53* locus (7372 bp) into the mouse striatum, and AsCpf1 expression was examined by immunofluorescence staining using an anti-AsCpf1 antibody in the brain tissues (Figure 5). At 21 days post AAV injection, we prepared serial brain sections and monitored EGFP expression under the fluorescence microscope to cut out EGFP-positive tissues from multiple slides. After pooling EGFP-positive brain tissues, the genomic DNAs were isolated for the T7E1 assays and targeted deep sequencing analyses (Figure 5B, bottom). Collectively, these data demonstrate that our H1 promoter-based CRISPR-AsCpf1 AAV system can induce indel mutations in vivo.

## 3. Discussion

Adeno-associated virus (AAV) vectors are well established for in vivo genome editing due to their favorable safety profile and efficient transduction. However, their limited packaging capacity presents a significant challenge for delivering multiplex CRISPR systems. In this study, we developed a CRISPR–AsCpf1 platform that leverages the intrinsic bidirectional transcriptional activity of the mouse H1 promoter, enabling multiplex genome editing from a single AAV vector. As genome-editing strategies increasingly incorporate advanced modalities such as base editors and prime editors [12,13], which require substantially larger vector payloads due to the inclusion of multiple functional domains, compact regulatory architectures are becoming increasingly important. In this context, our bidirectional H1 promoter–based design offers a practical strategy to minimize vector size while maintaining genome-editing functionality.

Although H1 promoter–based systems have previously been used to co-express shRNAs and protein-coding sequences [9], these approaches were not designed for AAV-mediated genome editing. Here, we demonstrate that an H1 promoter–based architecture can support multiplex genome editing and is compatible with both in vitro and in vivo applications. Notably, related approaches have been described in patent literature, including the use of the human H1 promoter to drive the expression of both guide RNA and Cas9 [14]. In contrast, the present study employs an optimized mouse H1 promoter to enable multiplex Cpf1-mediated genome editing from a single AAV vector.

Despite the compact size of the H1-based system, several limitations should be considered. Persistent nuclease expression from AAV vectors may increase the risk of off-target genome editing due to sustained activity, and this limitation should be carefully considered. Although we are aware of this potential risk, we selected the Cpf1 (Cas12a) system because it has been reported to exhibit higher genome-editing fidelity compared to Cas9 [15,16]. In addition, the current system exhibits lower AsCpf1 expression compared with conventional Pol II–driven dual-promoter architectures, resulting in modestly reduced indel efficiencies. As the H1 promoter is derived from regulatory elements associated with the *Parp2* locus, its activity may be influenced by tissue-specific transcriptional regulation, potentially leading to variable genome-editing efficiency across different cellular contexts. Furthermore, although EGFP expression in HepG2 cells suggests cross-species functionality, comprehensive validation in human cells remains to be performed.

Despite these limitations, the compact and flexible design of the bidirectional H1 promoter system offers significant advantages for size-constrained applications and may enable more selective or context-dependent genome editing. Future optimization strategies, including promoter engineering or incorporation of additional regulatory elements, may further improve the consistency and efficiency of the system. Notably, previous studies have shown that certain Pol III promoters can exhibit RNA Pol II activity [6], suggesting that engineered promoter variants may further enhance performance without increasing vector size.

In summary, this study establishes a compact, multiplex-capable H1 promoter–based CRISPR–AsCpf1 system that addresses key challenges in AAV-mediated genome-editing delivery and provides a foundation for further optimization toward in vivo multiplex genome-editing applications.

## 4. Materials and Methods

### 4.1. Cell Culture

NIH3T3 cells were kindly provided by Prof. Suwhan Chang (Asan Medical Center, Seoul, Republic of Korea). HEK293TA cells were purchased from GeneCopoeia (#LT008, GeneCopoeia, Rockville, MD, USA). HepG2 cells were kindly provided by Prof. Inki Kim (Asan Medical Center, Seoul, Republic of Korea). As described previously [17], primary mouse embryonic fibroblasts (MEFs) were isolated from E13.5 mouse embryos by mechanical dissociation followed by enzymatic digestion with 0.05% trypsin-EDTA and cultured under standard conditions. Cells were cultured in Dulbecco’s Modified Eagle Medium (DMEM; #SH30243.01, HyClone, Logan, UT, USA) supplemented with 10% fetal bovine serum (FBS; #F0600-050, Sigma-Aldrich, St. Louis, MO, USA) and 1% penicillin–streptomycin (#15140-122, Thermo Fisher Scientific, Waltham, MA, USA). Cells were maintained at 37 °C in a humidified incubator with 5% CO_2_.

### 4.2. Plasmid Construction

Five variants of the bidirectional H1 promoter (pLG010–pLG014) were generated to compare their effects on *Trp53* crRNA and AsCpf1 expression (Figure 1B,C and Figure S1). The initial promoter fragment was amplified from C57BL/6 mouse genomic DNA and inserted into pY010 (#69982, Addgene, Watertown, MA, USA) to generate pCG010. A *Trp53* crRNA sequence (Macrogen, Seoul, Republic of Korea) was cloned into this vector to obtain pCG010-*Trp53*. The resulting construct was then subcloned into the lentiviral backbone pY108 (#84739, Addgene, Watertown, MA, USA), which carries the AsCpf1–P2A–PuroR cassette, to produce pLG010-*Trp53* (Figure 1B, construct #3). Additional variants (pLG011–pLG014) were generated by PCR amplification using variant-specific primers.

To investigate the influence of the 5′ untranslated region (UTR) length of H1 RNA on crRNA transcription and indel activity, the UTR length was modified from 1 to 30 base pairs, generating variants V1 to V4 (Figure 1E). To compare genome-editing efficiency between the single-promoter (bidirectional H1) and dual-promoter (EFS and U6) systems, two types of lentiviral vectors targeting *Trp53* were constructed (Figure 2A). For AAV delivery using the CRISPR–AsCpf1 system, the pAG013 v2 vector was constructed by inserting the bidirectional H1 promoter and the AsCpf1 coding sequence into the pX601 AAV vector (#61591, Addgene, Watertown, MA, USA). crRNAs targeting *Trp53, Trp53/Pten*, and *Trp53/Pten/Nf1* were individually cloned into the BsmBI site of the pAG013 v2 vector (Figure 4). The following target sequences were used: 5′-TTT CGC CAC AGC GTG GTG GTA CCT-3′ for *Trp53*; 5′-TGC TAA CGA TCT CTT TGA TGA TG-3′ for *Pten*; and 5′-CCA CAT GCT TTA GGC ACT AAC CT-3′ for *Nf1*. To minimize AAV vector size under packaging constraints, the impact of poly(A) tail length on indel frequency was assessed using SV40 poly(A) (122 bp) and bovine growth hormone (bGH) poly(A) (208 bp) sequences (Figure S3A).

### 4.3. Lentivirus Packaging and Puromycin Selection

For lentivirus production, HEK293TA cells were seeded at 70–80% confluency in 60 mm dishes and transfected with 1 μg of plasmid DNA consisting of the transfer vector (pLenti), packaging plasmid (psPAX2; Addgene #12260), and envelope plasmid (pMD2.G; Addgene #12259, VSV-G) at a ratio of 5:2:4 using Lipofectamine (#3138621, Thermo Fisher Scientific, Waltham, MA, USA) and Plus reagent (#11514015, Thermo Fisher Scientific, Waltham, MA, USA) according to the manufacturer’s instructions. At 24 h post-transfection, culture media containing lentiviral particles were collected and used for infection. For puromycin selection, NIH3T3 cells were seeded at 5 × 10^4^ cells per well in 24-well plates and infected with 50 μL of lentiviral supernatant. After 24 h, infected cells were treated with puromycin (2 μg/mL; #P8833-10MG, Sigma-Aldrich, St. Louis, MO, USA) to eliminate non-infected cells. Selection media were replaced every 3 days for 2 weeks. Genomic DNA from puromycin-selected cells was isolated and used for T7 endonuclease I (T7E1) assays and deep sequencing analysis.

### 4.4. AAV Packaging

For AAV production, HEK293TA cells were seeded at 70–80% confluency in 100 mm dishes and transfected the following day with 24 μg of plasmid DNA (pAAV:pRC-DJ:pHelper = 1:1:2) using Lipofectamine and Plus reagent. At 72 h post-transfection, cells were harvested and lysed by three freeze–thaw cycles. AAV particles were collected by centrifugation at 3000× *g* for 15 min at 4 °C and concentrated using Amicon Ultra centrifugal filters (#0000246467, Millipore, Burlington, MA, USA). This procedure yielded partially purified AAV preparations. AAV titers were determined by quantitative real-time PCR using the AAVpro^®^ Titration Kit (Takara Bio Inc., #6233, Shiga, Japan), according to the manufacturer’s instructions. The resulting viral titers ranged from approximately 10^9^ to 10^10^ vg/mL, depending on the preparation conditions. Purified AAV-DJ was also obtained from the IBS Virus Facility (Daejeon, Republic of Korea). AAV-DJ was used due to its high transduction efficiency across multiple cell types, as previously reported [18].

### 4.5. T7 Endonuclease I (T7E1) Assay

Genomic DNA was isolated from cells and tissues using a standard phenol–chloroform extraction method. Briefly, samples were lysed in lysis buffer containing proteinase K and then incubated at 55 °C overnight until complete digestion. The lysates were then mixed with a 500 μL volume of phenol–chloroform (1:1) solution and centrifuged at 3000 rpm for 10 min to achieve phase separation. The aqueous phase was subsequently extracted with a 500 μL volume of chloroform and centrifuged at 3000 rpm for 10 min. DNA was precipitated by the addition of a 750 μL volume of 100% ethanol and gently inverted to facilitate DNA aggregation. The DNA pellet was collected with a sterile pipette tip and resuspended in nuclease-free water. Target genomic regions were amplified by PCR and purified using a PCR purification kit (#A27335, Thermo Fisher Scientific, Waltham, MA, USA). Purified PCR products (500 ng) were denatured at 95 °C for 5 min and then slowly cooled to allow reannealing. T7E1 enzyme (5 U; #M0302L, New England Biolabs, Ipswich, MA, USA) was added, and samples were incubated at 37 °C for 15 min. Digested products were resolved on 2% agarose gels, and indel frequencies were estimated based on the relative intensities of cleaved and uncleaved bands. The PCR primers used were as follows: *Trp53*, 5′-ACA TGA CGG AGG TCG TGA GA-3′ and 5′-CAT CAG TCT AGG CTG GAG TCA A-3′; *Pten*, 5′-CTC TGG CTG CTG AGG AGA AG-3′ and 5′-CAA GGA GGC ATG AGT TCC GT-3′; *Nf1*, 5′-TTC GAG TGA TAG CTG TGT GGC-3′ and 5′-GAA TTC CTT CAA AAA CCC AGA TGT-3′. Indel mutations were detected using T7E1 assays, which cleave DNA heteroduplexes at mismatched base pairs [19].

### 4.6. Deep Sequencing Analysis of Genome Editing

Deep sequencing was performed to quantify genome-editing efficiencies at target loci. Genomic DNA was isolated from gene-edited cells or brain samples. For multiplex targeting experiments (Figure 4B,C), samples were derived from multiple regions, whereas for all other experiments, samples were derived from a single region. Genomic regions containing the target sites were first amplified using locus-specific PCR primer pairs previously employed for the T7E1 assays. The resulting amplicons then underwent two additional PCR steps to incorporate Illumina overhang adapter sequences and index sequences (Tables S1 and S2). These indexed PCR products were purified using a PCR purification kit (#A27335, Thermo Fisher Scientific, Waltham, MA, USA). All libraries were normalized to a final concentration of 12 ng/μL, pooled at equimolar ratios, and subjected to deep sequencing. Sequencing was performed on an Illumina iSeq 100 System using paired-end reads. Indel frequencies were determined by aligning reads to the mouse reference genome using Cas-Analyzer (version1.0), a web-based tool within the CRISPR RGEN Tools software [20]. Genome-editing efficiency was calculated as the percentage of reads containing indel mutations relative to the total number of aligned reads. Targeted deep sequencing of amplicons was performed at a high depth, with approximately 7000–8000 reads per sample, which provides sufficiently reliable detection of indel events.

### 4.7. Western Blot Analysis

To assess AsCpf1 expression following lentiviral or AAV infection, NIH3T3 cells were washed with ice-cold phosphate-buffered saline (#LB001-02, WELGENE Inc., Daejeon, Republic of Korea) and lysed directly in SDS sample buffer (#LC2676, Thermo Fisher Scientific, Waltham, MA, USA). Protein concentrations were determined using a BCA assay (#23225, Thermo Fisher Scientific, Waltham, MA, USA). Equal amounts of protein were separated on 10% SDS–PAGE gels and transferred to PVDF membranes (#IPVH08100, Millipore, Burlington, MA, USA). Membranes were blocked with 5% skim milk in Tris-buffered saline containing 0.1% Tween 20 and probed with primary antibodies against AsCpf1 (1:1000, #38105, Cell Signaling Technology, Danvers, MA, USA) and β-actin (1:5000, #NB600-501, Novus Biologicals, Centennial, CO, USA), followed by HRP-conjugated secondary anti-rabbit antibodies (#sc-2004, Santa Cruz Biotechnology, Dallas, TX, USA) and HRP-conjugated secondary anti-mouse antibodies (#sc-2005, Santa Cruz Biotechnology, Dallas, TX, USA). Signals were detected using a chemiluminescence detection system (Bio-Rad, Hercules, CA, USA).

### 4.8. Stereotaxic AAV Injection and Tissue Processing

Male C57BL/6 mice (8 weeks old, 22–25 g, *n* = 9) were anesthetized with isoflurane and secured in a stereotaxic apparatus (#JD-SI-02MR, Jeungdo Bio & Plant Co. Ltd., Seoul, Republic of Korea). PBS or AAV (1–2 μL) was injected into the right striatum at a rate of 0.2 μL/min using a Hamilton syringe (#HAM80075, Sigma-Aldrich, St. Louis, MO, USA) at the following coordinates relative to bregma: anteroposterior +0.2 mm, mediolateral +2.0 mm, dorsoventral −3.5 mm. After injection, the needle was held in place for 5 min and then withdrawn slowly to minimize backflow. Of these, three mice were used for the EGFP expression experiment, and six mice were used for the in vivo genome-editing experiment. For tissue processing, mice were perfused intracardially with saline followed by 4% paraformaldehyde in 0.1 M phosphate buffer. Brains were post-fixed in 4% paraformaldehyde overnight at 4 °C, cryoprotected in 30% sucrose, frozen, and coronally sectioned at 40 μm using a cryostat (CM1080; Leica, Wetzlar, Germany). Sections were stored in PBS at 4 °C until further analysis.

### 4.9. Immunofluorescent Staining

Immunofluorescent staining was performed using standard procedures. Free-floating sections were washed with PBS and blocked in 5% normal goat serum (#PCN5000, Thermo Fisher Scientific, Waltham, MA, USA) containing 0.3% Triton X-100 for 1 h at room temperature. Sections were then incubated overnight at 4 °C with primary antibodies against AsCpf1 (1:400; #19984, Cell Signaling Technology, Danvers, MA, USA), followed by incubation with Alexa Fluor 594-conjugated secondary antibodies (1:300; #A11012, Thermo Fisher Scientific, Waltham, MA, USA) for 1 h at room temperature. Sections were mounted with VectaShield mounting medium (#H-1000-10, Vector Laboratories, Burlingame, CA, USA) and imaged using a confocal laser scanning microscope (LSM 880; Carl Zeiss, Oberkochen, Germany). GFP signals were detected by direct fluorescence without antibody staining.

## Data Availability

The data presented in this study are available on request from the corresponding authors.

## Supplementary Materials

The following supporting information can be downloaded at: https://www.mdpi.com/article/10.3390/ijms27104162/s1.
