# Supplementary material for: CRISPR-Cpf1-Mediated Gene-Editing System Based on a Single Bidirectional Promoter
Source: Int J Mol Sci. 2026 May 7;27(10):4162. doi: 10.3390/ijms27104162 (PMC13206784; doi:10.3390/ijms27104162)
Supplement: Supplementary file 1 [file ijms-27-04162-s001.zip › ijms-4208964-supplementary materials.pptx]

## Slide 1
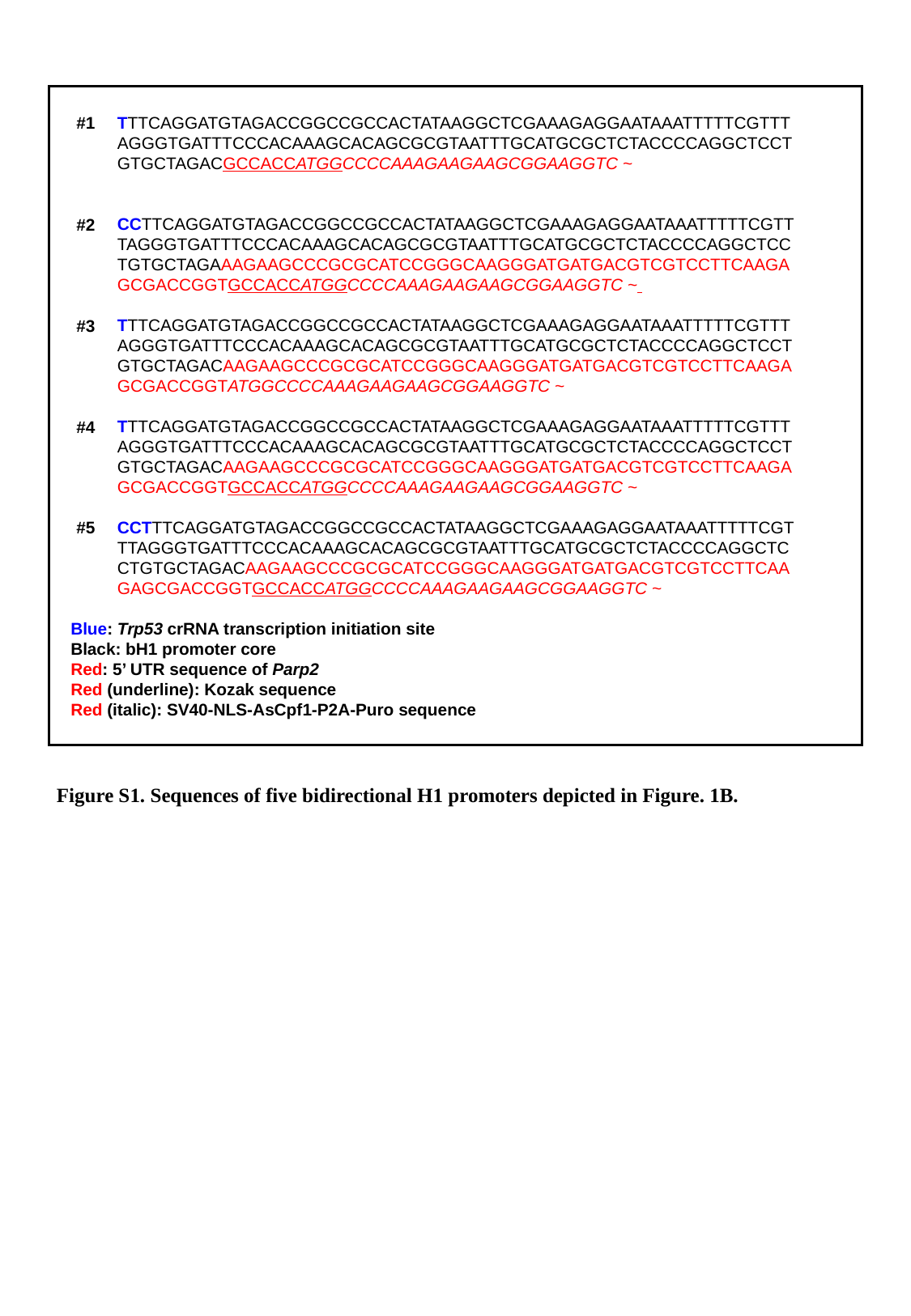

TTTCAGGATGTAGACCGGCCGCCACTATAAGGCTCGAAAGAGGAATAAATTTTTCGTTTAGGGTGATTTCCCACAAAGCACAGCGCGTAATTTGCATGCGCTCTACCCCAGGCTCCTGTGCTAGACGCCACCATGGCCCCAAAGAAGAAGCGGAAGGTC ~
CCTTCAGGATGTAGACCGGCCGCCACTATAAGGCTCGAAAGAGGAATAAATTTTTCGTTTAGGGTGATTTCCCACAAAGCACAGCGCGTAATTTGCATGCGCTCTACCCCAGGCTCCTGTGCTAGAAAGAAGCCCGCGCATCCGGGCAAGGGATGATGACGTCGTCCTTCAAGAGCGACCGGTGCCACCATGGCCCCAAAGAAGAAGCGGAAGGTC ~
TTTCAGGATGTAGACCGGCCGCCACTATAAGGCTCGAAAGAGGAATAAATTTTTCGTTTAGGGTGATTTCCCACAAAGCACAGCGCGTAATTTGCATGCGCTCTACCCCAGGCTCCTGTGCTAGACAAGAAGCCCGCGCATCCGGGCAAGGGATGATGACGTCGTCCTTCAAGAGCGACCGGTATGGCCCCAAAGAAGAAGCGGAAGGTC ~
TTTCAGGATGTAGACCGGCCGCCACTATAAGGCTCGAAAGAGGAATAAATTTTTCGTTTAGGGTGATTTCCCACAAAGCACAGCGCGTAATTTGCATGCGCTCTACCCCAGGCTCCTGTGCTAGACAAGAAGCCCGCGCATCCGGGCAAGGGATGATGACGTCGTCCTTCAAGAGCGACCGGTGCCACCATGGCCCCAAAGAAGAAGCGGAAGGTC ~
CCTTTCAGGATGTAGACCGGCCGCCACTATAAGGCTCGAAAGAGGAATAAATTTTTCGTTTAGGGTGATTTCCCACAAAGCACAGCGCGTAATTTGCATGCGCTCTACCCCAGGCTCCTGTGCTAGACAAGAAGCCCGCGCATCCGGGCAAGGGATGATGACGTCGTCCTTCAAGAGCGACCGGTGCCACCATGGCCCCAAAGAAGAAGCGGAAGGTC ~
 Blue: Trp53 crRNA transcription initiation site
 Black: bH1 promoter core
 Red: 5’ UTR sequence of Parp2
 Red (underline): Kozak sequence
 Red (italic): SV40-NLS-AsCpf1-P2A-Puro sequence
#1
#2
#3
#4
#5
Figure S1. Sequences of five bidirectional H1 promoters depicted in Figure. 1B.

## Slide 2
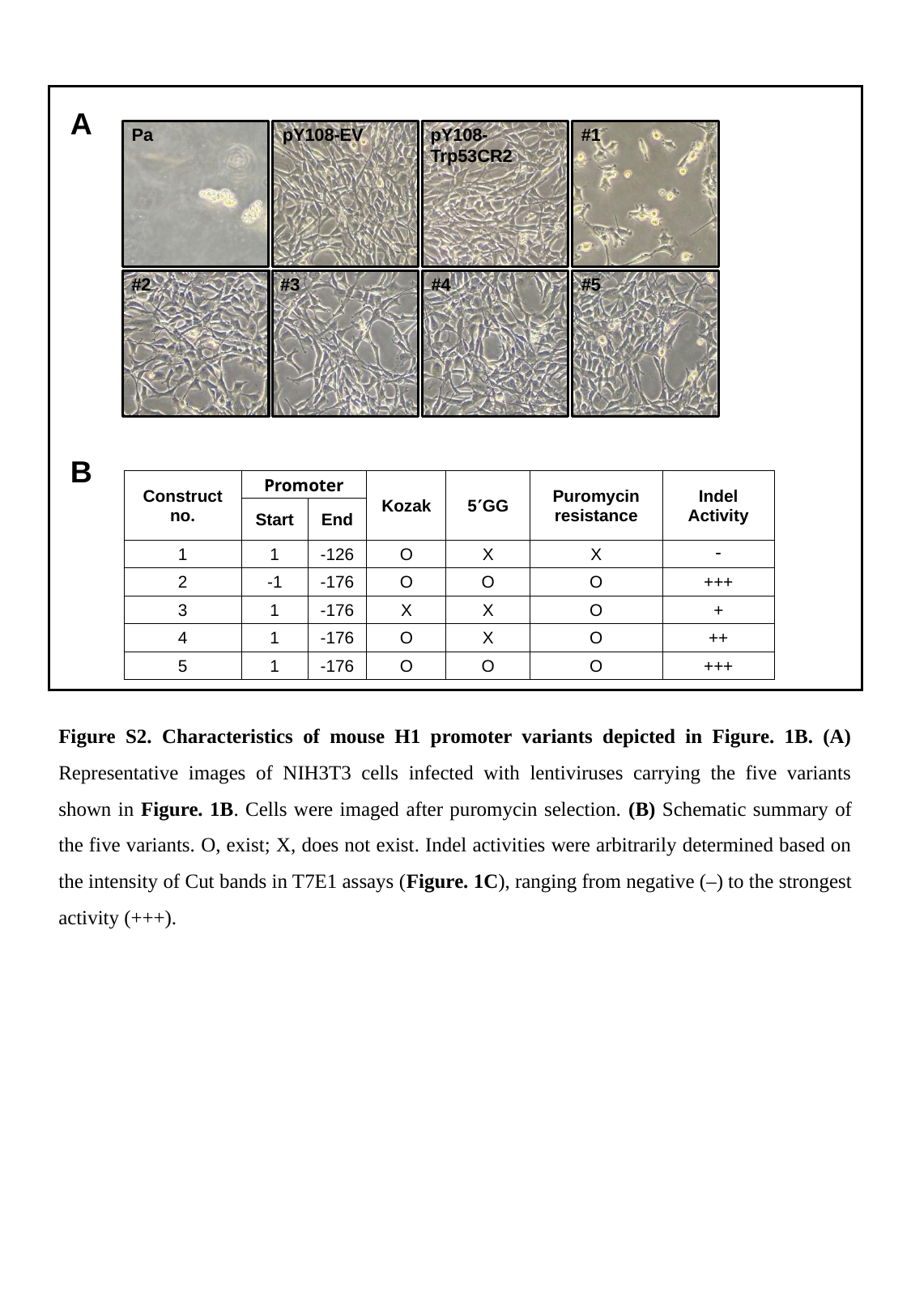

A
Pa
pY108-EV
pY108-Trp53CR2
#1
#2
#3
#4
#5
B
| Construct no. | Promoter | | Kozak | 5¢GG | Puromycin resistance | Indel Activity |
| --- | --- | --- | --- | --- | --- | --- |
| | Start | End | | | | |
| 1 | 1 | -126 | O | X | X | − |
| 2 | -1 | -176 | O | O | O | +++ |
| 3 | 1 | -176 | X | X | O | + |
| 4 | 1 | -176 | O | X | O | ++ |
| 5 | 1 | -176 | O | O | O | +++ |
Figure S2. Characteristics of mouse H1 promoter variants depicted in Figure. 1B. (A) Representative images of NIH3T3 cells infected with lentiviruses carrying the five variants shown in Figure. 1B. Cells were imaged after puromycin selection. (B) Schematic summary of the five variants. O, exist; X, does not exist. Indel activities were arbitrarily determined based on the intensity of Cut bands in T7E1 assays (Figure. 1C), ranging from negative (–) to the strongest activity (+++).

## Slide 3
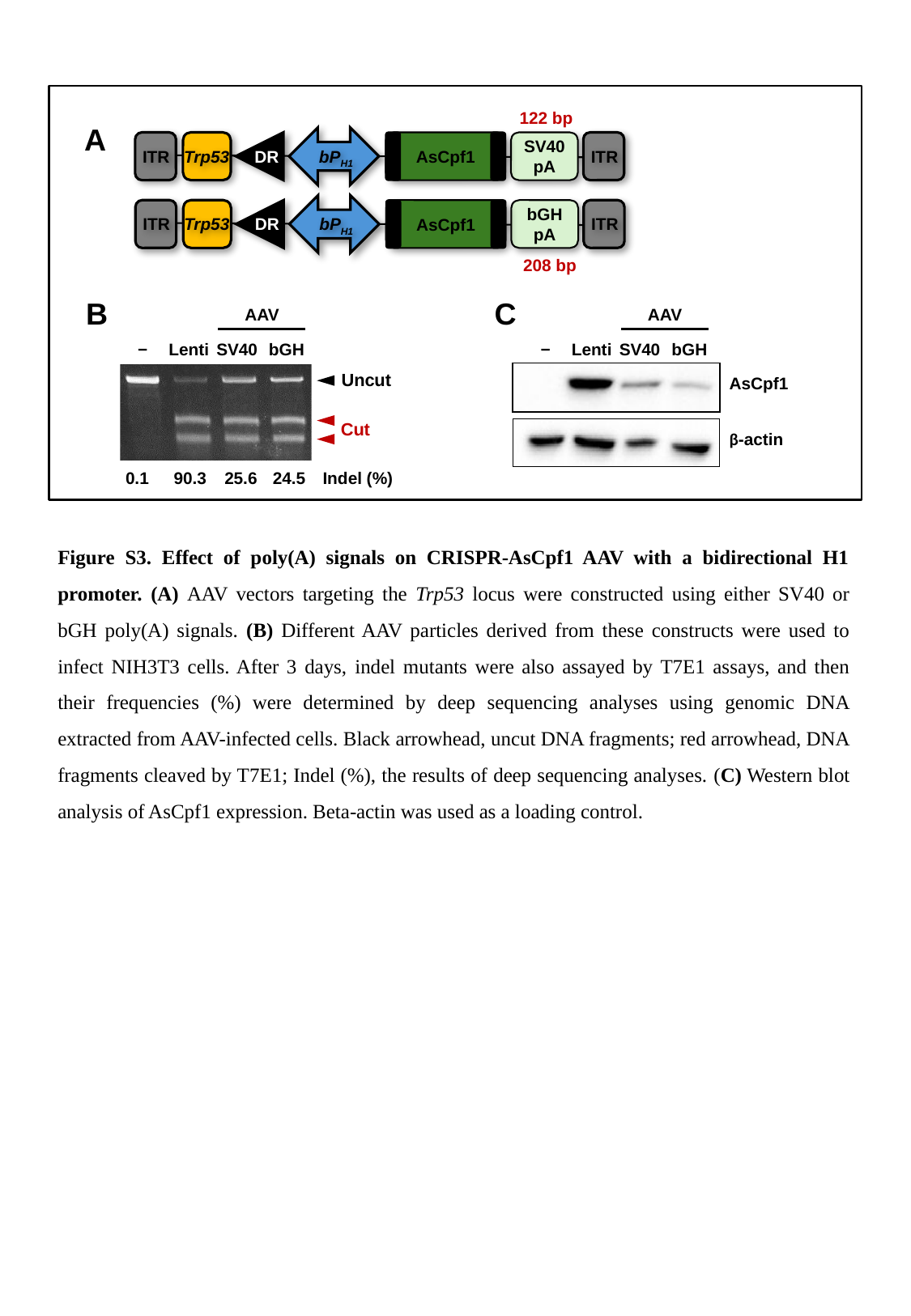

122 bp
A
AsCpf1
SV40
pA
ITR
Trp53
DR
bPH1
ITR
AsCpf1
bGH
pA
ITR
Trp53
DR
bPH1
ITR
208 bp
B
C
AAV
−
Lenti
SV40
bGH
AAV
−
Lenti
SV40
bGH
 Uncut
AsCpf1
 Cut
β-actin
0.1
90.3
25.6
24.5
Indel (%)
Figure S3. Effect of poly(A) signals on CRISPR-AsCpf1 AAV with a bidirectional H1 promoter. (A) AAV vectors targeting the Trp53 locus were constructed using either SV40 or bGH poly(A) signals. (B) Different AAV particles derived from these constructs were used to infect NIH3T3 cells. After 3 days, indel mutants were also assayed by T7E1 assays, and then their frequencies (%) were determined by deep sequencing analyses using genomic DNA extracted from AAV-infected cells. Black arrowhead, uncut DNA fragments; red arrowhead, DNA fragments cleaved by T7E1; Indel (%), the results of deep sequencing analyses. (C) Western blot analysis of AsCpf1 expression. Beta-actin was used as a loading control.

## Slide 4
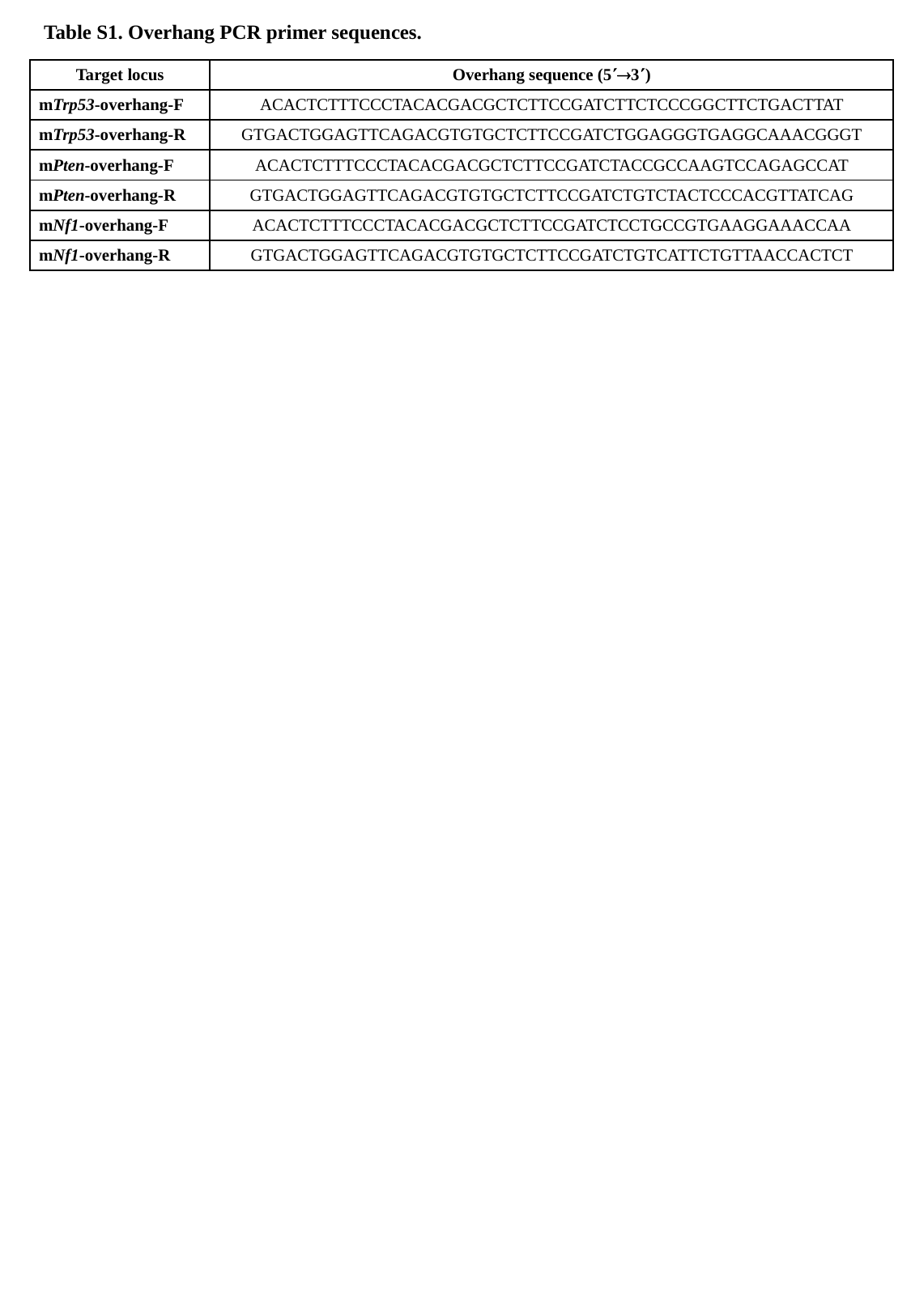

Table S1. Overhang PCR primer sequences.
| Target locus | Overhang sequence (53′) |
| --- | --- |
| mTrp53-overhang-F | ACACTCTTTCCCTACACGACGCTCTTCCGATCTTCTCCCGGCTTCTGACTTAT |
| mTrp53-overhang-R | GTGACTGGAGTTCAGACGTGTGCTCTTCCGATCTGGAGGGTGAGGCAAACGGGT |
| mPten-overhang-F | ACACTCTTTCCCTACACGACGCTCTTCCGATCTACCGCCAAGTCCAGAGCCAT |
| mPten-overhang-R | GTGACTGGAGTTCAGACGTGTGCTCTTCCGATCTGTCTACTCCCACGTTATCAG |
| mNf1-overhang-F | ACACTCTTTCCCTACACGACGCTCTTCCGATCTCCTGCCGTGAAGGAAACCAA |
| mNf1-overhang-R | GTGACTGGAGTTCAGACGTGTGCTCTTCCGATCTGTCATTCTGTTAACCACTCT |

## Slide 5
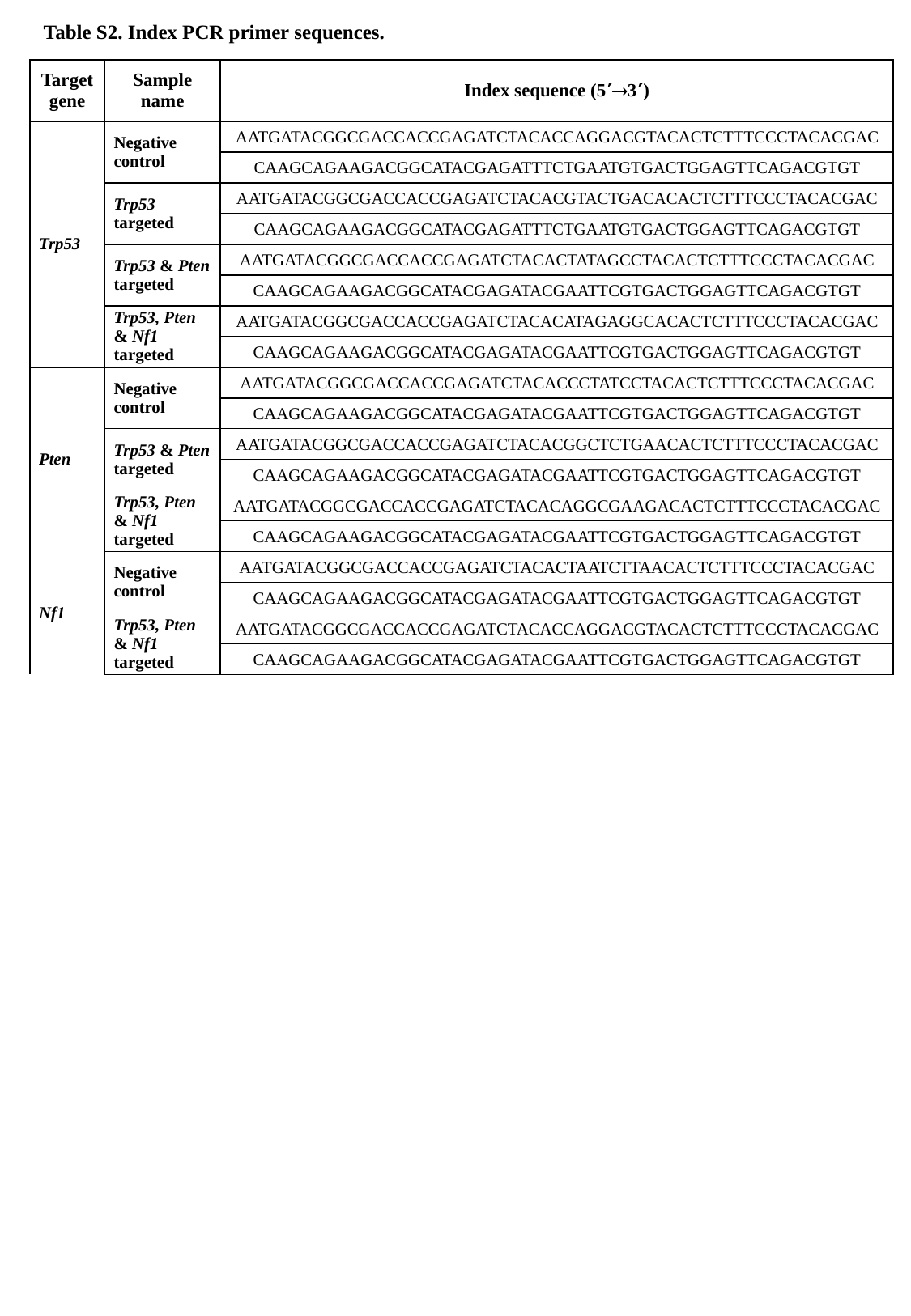

Table S2. Index PCR primer sequences.
| Target gene | Sample name | Index sequence (53′) |
| --- | --- | --- |
| Trp53 | Negative control | AATGATACGGCGACCACCGAGATCTACACCAGGACGTACACTCTTTCCCTACACGAC |
| | | CAAGCAGAAGACGGCATACGAGATTTCTGAATGTGACTGGAGTTCAGACGTGT |
| | Trp53 targeted | AATGATACGGCGACCACCGAGATCTACACGTACTGACACACTCTTTCCCTACACGAC |
| | | CAAGCAGAAGACGGCATACGAGATTTCTGAATGTGACTGGAGTTCAGACGTGT |
| | Trp53 & Pten targeted | AATGATACGGCGACCACCGAGATCTACACTATAGCCTACACTCTTTCCCTACACGAC |
| | | CAAGCAGAAGACGGCATACGAGATACGAATTCGTGACTGGAGTTCAGACGTGT |
| | Trp53, Pten & Nf1 targeted | AATGATACGGCGACCACCGAGATCTACACATAGAGGCACACTCTTTCCCTACACGAC |
| | | CAAGCAGAAGACGGCATACGAGATACGAATTCGTGACTGGAGTTCAGACGTGT |
| Pten | Negative control | AATGATACGGCGACCACCGAGATCTACACCCTATCCTACACTCTTTCCCTACACGAC |
| | | CAAGCAGAAGACGGCATACGAGATACGAATTCGTGACTGGAGTTCAGACGTGT |
| | Trp53 & Pten targeted | AATGATACGGCGACCACCGAGATCTACACGGCTCTGAACACTCTTTCCCTACACGAC |
| | | CAAGCAGAAGACGGCATACGAGATACGAATTCGTGACTGGAGTTCAGACGTGT |
| | Trp53, Pten & Nf1 targeted | AATGATACGGCGACCACCGAGATCTACACAGGCGAAGACACTCTTTCCCTACACGAC |
| | | CAAGCAGAAGACGGCATACGAGATACGAATTCGTGACTGGAGTTCAGACGTGT |
| Nf1 | Negative control | AATGATACGGCGACCACCGAGATCTACACTAATCTTAACACTCTTTCCCTACACGAC |
| | | CAAGCAGAAGACGGCATACGAGATACGAATTCGTGACTGGAGTTCAGACGTGT |
| | Trp53, Pten & Nf1 targeted | AATGATACGGCGACCACCGAGATCTACACCAGGACGTACACTCTTTCCCTACACGAC |
| | | CAAGCAGAAGACGGCATACGAGATACGAATTCGTGACTGGAGTTCAGACGTGT |
